# A Novel *SCN5A* Mutation Associated with Drug Induced Brugada Type ECG

**DOI:** 10.1371/journal.pone.0161872

**Published:** 2016-08-25

**Authors:** Isik Turker, Takeru Makiyama, Matteo Vatta, Hideki Itoh, Takeshi Ueyama, Akihiko Shimizu, Tomohiko Ai, Minoru Horie

**Affiliations:** 1 Krannert Institute of Cardiology, Indiana University, Indianapolis, IN, United States of America; 2 Cardiovascular Medicine, Kyoto University Graduate School of Medicine, Kyoto, Japan; 3 Department of Cardiovascular Genetics, Indiana University, Indianapolis, IN, United States of America; 4 Cardiovascular and Respiratory Medicine, Shiga Univ. School of Medicine, Otsu, Japan; 5 Cardiology, Yamaguchi University School of Medicine, Yamaguchi, Japan; 6 Molecular Pathogenesis, Medical Research Institute, Tokyo Medical and Dental University, Tokyo, Japan; Georgia State University, UNITED STATES

## Abstract

**Background:**

Class IC antiarrhythmic agents may induce acquired forms of Brugada Syndrome. We have identified a novel mutation in *SCN5A*, the gene that encodes the α-subunit of the human cardiac sodium channel (hNa_v_1.5), in a patient who exhibited Brugada- type ECG changes during pharmacotherapy of atrial arrhythmias.

**Objective:**

To assess whether the novel mutation p.V1328M can cause drug induced Brugada Syndrome.

**Methods:**

Administration of pilsicainide, a class IC antiarrhythmic agent, caused Brugada- type ST elevation in a 66-year-old Japanese male who presented with paroxysmal atrial fibrillation (PAF), type I atrial flutter and inducible ventricular fibrillation (VF) during electrophysiological study. Genetic screening using direct sequencing identified a novel *SCN5A* variant, p.V1328M. Electrophysiological parameters of WT and p.V1328M and their effects on drug pharmacokinetics were studied using the patch-clamp method.

**Results:**

Whole-cell sodium current densities were similar for WT and p.V1328M channels. While p.V1328M mutation did not affect the voltage-dependence of the activation kinetics, it caused a positive shift of voltage-dependent steady-state inactivation by 7 mV. The tonic block in the presence of pilsicainide was similar in WT and p.V1328M, when sodium currents were induced by a low frequency pulse protocol (q15s). On the contrary, p.V1328M mutation enhanced pilsicainide induced use-dependent block at 2 Hz. (*Ki*: WT, 35.8 μM; V1328M, 19.3 μM).

**Conclusion:**

Our study suggests that a subclinical *SCN5A* mutation, p.V1328M, might predispose individuals harboring it to drug-induced Brugada Syndrome.

## Introduction

Pilsicainide, a class IC antiarrhythmic agent, has been widely used in Japan for the diagnosis and treatment of atrial arrhythmias [1, 2], as well as in the diagnosis of Brugada Syndrome [3, 4]. It is hypothesized that antiarrhythmic agents can result in or exaggerate the existing right precordial ST elevation by further reducing sodium currents that are already affected by variants of *SCN5A*, the gene that encodes the α-subunit of cardiac sodium channels (hNa_v_1.5). Among various antiarrhythmic agents, those of class IC cause the most pronounced ST change by strong blockage of the sodium channels, which may result in acquired forms of Brugada Syndrome [5] or worsening of arrhythmias in patients with Brugada Syndrome [6, 7]. *SCN5A* variants may cause unpredictable hNa_v_1.5 response to antiarrythmic agents. For example, Itoh *et al*. reported that the *SCN5A* variant p.N406S identified in Brugada Syndrome patients enhanced use-dependent block of sodium currents with quinidine, a class IA antiarrythmic agent, but the same variant abolished the block with pilsicainide [8]. On the other hand, we demonstrated that a common *SCN5A* single nucleotide polymorphism can enhance use-dependent block of hNav1.5 with quinidine, and with the class IC agent flecainide [9].

In this study, we report the case of a 66-year-old Japanese male who developed pilsicainide induced Brugada-type ECG during pharmacotherapy of atrial arrhythmias. Genetic screening identified a novel c.3982G>A variant in *SCN5A*, resulting in substitution of methionine for valine at position 1328 (p.V1328M) located in the cytoplasmic loop between the segment 4 and segment 5 of domain III of the protein. Biophysical modification of the hNa_v_1.5 by p.V1328M and its effects on sodium channel pharmacokinetics were studied using the patch-clamp method.

## Materials and Methods

### Genetic screening and mutagenesis

Genetic analysis was performed according to the guidelines approved by the Institutional Ethics Committee at Kyoto University. A written informed consent was obtained from the patient and screened family members. Genomic DNA was isolated from leukocyte nuclei using the QIAmp DNA Blood Midi Kit (QIAGEN GmbH, Hilden, Germany). Genetic screening for *SCN5A* variants was done using denaturing high-performance liquid chromatography (DHPLC: WAVE System Model 3500, Transgenomic, Omaha, NE). Direct DNA sequencing was done with the ABI PRISM3100 DNA Sequencer (Applied Biosystems, Wellesley, MA).

Site-directed mutagenesis was performed with the QuikChange Site-Directed Mutagenesis Kit (Stratagene, La Jolla, CA) using a vector containing the WT-*SCN5A-Q1077* (NM_000335.4) as a template. Mutant p.V1328M-*SCN5A* (hencefore V1328M) clones were verified by sequencing to ensure the presence of the variant and the absence of other substitutions introduced by the DNA polymerase.

### Cell culture and transient expression of WT and V1328M

Transient transfection of HEK-293 cells (ATCC, CRL-1573) with WT and V1328M-*SCN5A* was performed as described elsewhere [9]. Briefly, naïve HEK-293 cells were co-transfected with the plasmid pcDNA3.1 containing either WT or V1328M-*SCN5A* along with pEGFP-C3 (Clontech, Mountain View, CA) encoding green fluorescent protein, using Lipofectamine 2000 transfection reagent (Qiagen, Valencia, CA). The cells were incubated at 37°C for 2–3 days before use.

### Patch-clamp experiments

Patch-clamp experiments were performed as described previously [9] using Axopatch 200A amplifier and pClamp 9 software (Axon Instruments, Sunnyvale, CA). Whole-cell recordings were carried out at 22°C. Series resistance was 2–5 MΩ. Currents were filtered at 10 kHz with a built-in four-pole Bessel filter and digitized by a computer (Dell Optiplex GX280) at 20 kHz. The pipette solution contained 10 NaF, 110 CsF, 20 CsCl, 10 EGTA, and 10 HEPES (in mM, pH 7.35 with CsOH). Bath solution contained 145 NaCl, 4 KCl, 1 MgCl_2_, 1.8 CaCl_2_, 10 HEPES, and 10 glucose (in mM, pH 7.35 with NaOH). The estimated liquid junction potential was 8.1 mV and membrane potentials were corrected for it. Pilsicainide was kindly provided by Daiichi Sankyo Co., LTD. (Tokyo, Japan). Whole-cell currents were analyzed using the Clampfit (Axon Instruments, Sunnyvale, CA) and Igor softwares (Wavemetrics, Lake Oswego, OR). Data were presented as mean ± SE. *t*-Test was used for statistical analysis.

## Results

### Clinical Observations

**Fig 1:**
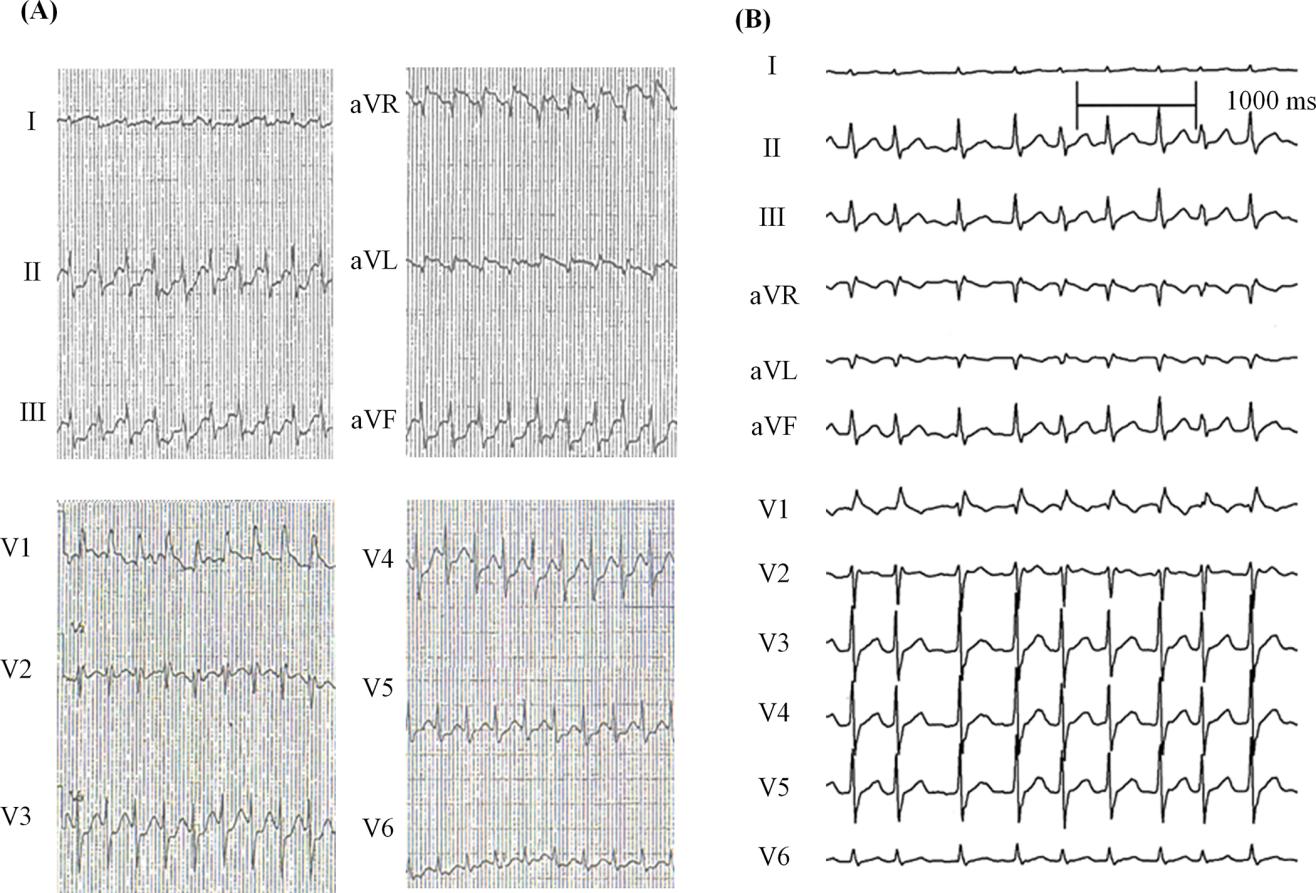
ECG phenotypes. (A) Type I atrial flutter. (B) PAF.

**Fig 2:**
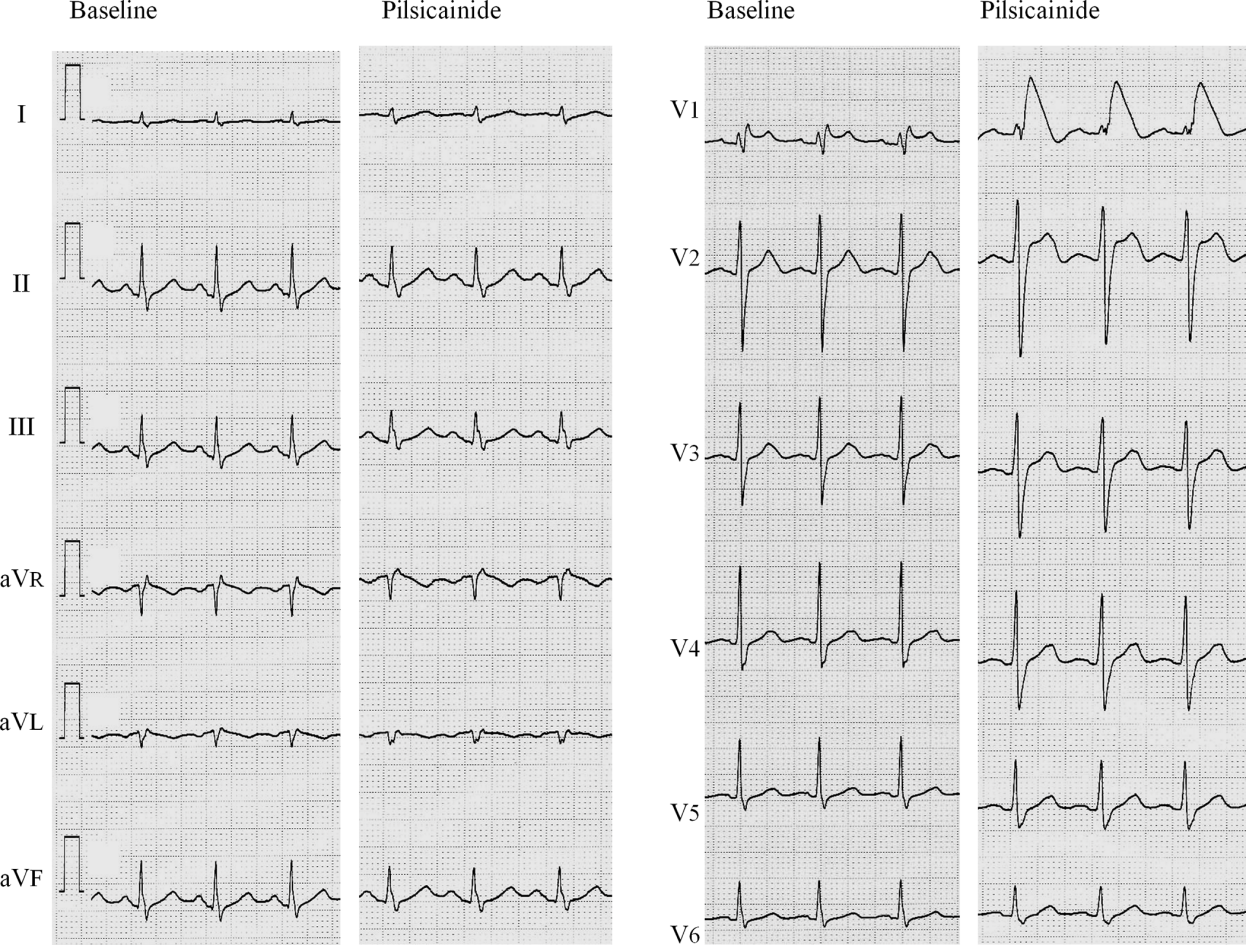
ECG phenotypes on pilsicainide. Left panels show baseline ECGs and the right panels show the ECGs after intravenous injection of pilsicainide (0.8 mg/kg).

The patient was a 66-year-old Japanese male with history of type I atrial flutter and PAF (Fig 1A and 1B). His baseline ECG showed normal sinus rhythm and incomplete right bundle branch block with mild ST elevation in lead V_1_ (Fig 1C). He had been treated with disopyramide, a class IA antiarrhythmic agent, and verapamil. He had no personal or family history of syncope or sudden cardiac death. His echocardiogram was normal. Three years into the above-mentioned antiarrythmic treatment, atrial arrhythmias recurred necessitating DC cardioversion. Post-cardioversion administration of pilsicainide caused marked coved type ST elevation in lead V_1_ and saddle-back type ST elevation in lead V_2_ (Fig 2).

Electrophysiological study (EPS) was performed to evaluate for arrhythmia substrates. During EPS, type I atrial flutter was induced and cavotricuspid isthmus ablation was performed. Furthermore, programmed stimulation induced VF (S1 Fig). The patient was discharged after discontinuation of all antiarrhythmic agents.

### Genetic Screening

**Fig 3:**
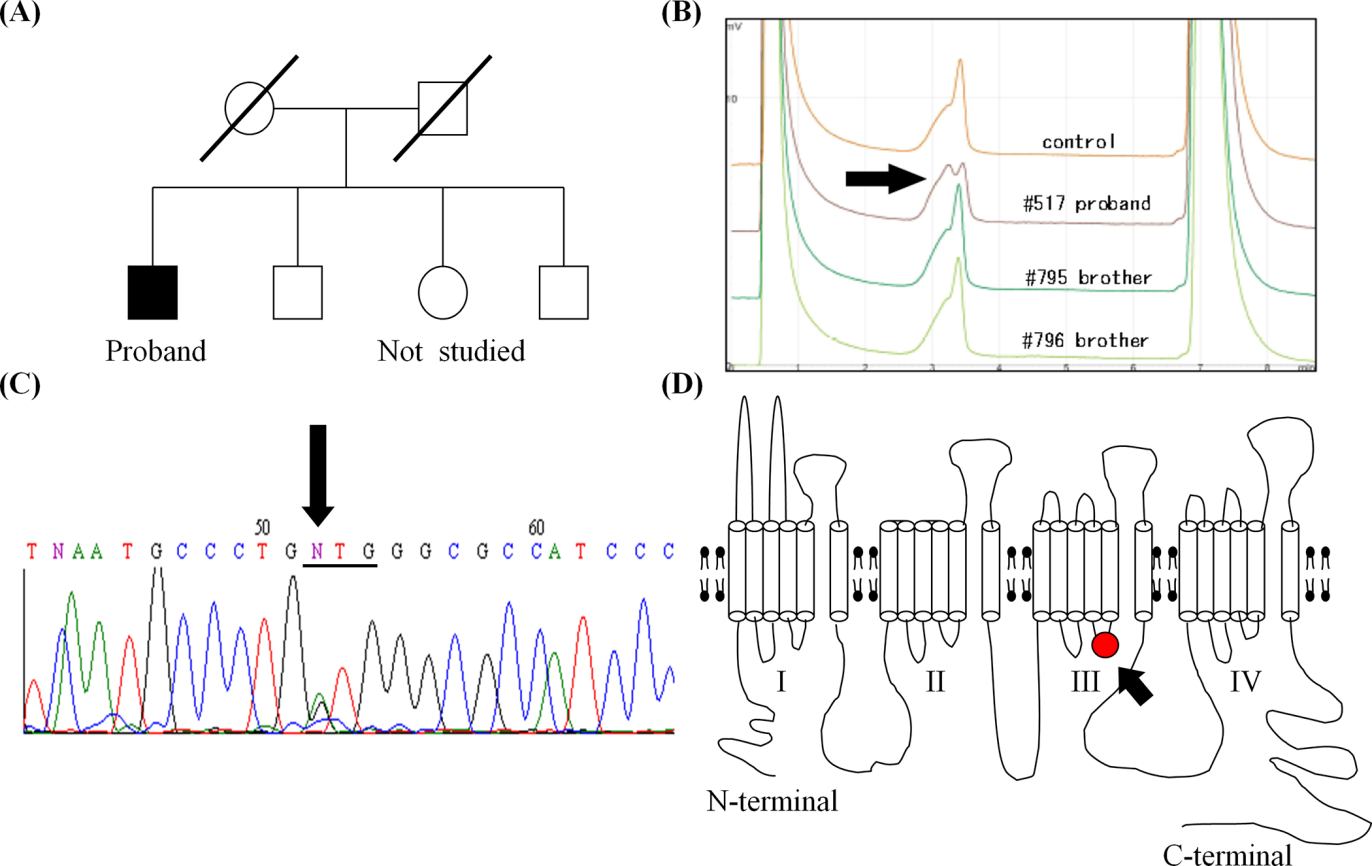
Genetic analysis. (A) Family tree. The filled square indicates the proband. (B) Abnormal migration pattern identified with DHPLC in the proband. (C) A nucleotide change (3982G>A) identified in the exon 23 of *SCN5A* (black arrow). (D) Schematic illustration of the SCN5A structure. The red closed circle with the arrowhead indicates the location of p.V1328M mutation.

Genetic screening revealed an aberrant band in the exon 23 of *SCN5A* (NM_198056) in the proband, but not in his two siblings (Fig 3A and 3B). Direct sequencing identified a nucleotide change, c.3982G>A, resulting in the substitution of methionine for valine at residue 1328 (p.V1328M; Fig 3C and 3D). This variant could not be found in any database we searched, including a local Japanese database (110 subjects, 220 alleles), the single nucleotide polymorphism database (dbSNP, build 146) [10], the 1000 Genomes Browser database, phase 3 (2,500 subjects) [11], the NHLBI Exome Sequencing Project (ESP) database (6,500 subjects) [12], and the Exome Aggregation Consortium (ExAC) (60,312 subjects). These results suggest that *SCN5A* p.V1328M is not a common variant. In addition, conservation analysis with the UCSC Genome Browser showed that the V1328 residue is highly conserved in mammals and chicken, but substituted by isoleucine in frog and zebrafish [13]. No other *SCN5A* variants were found in this patient.

### Basal electrophysiological parameters of V1328M-SCN5A

**Fig 4:**
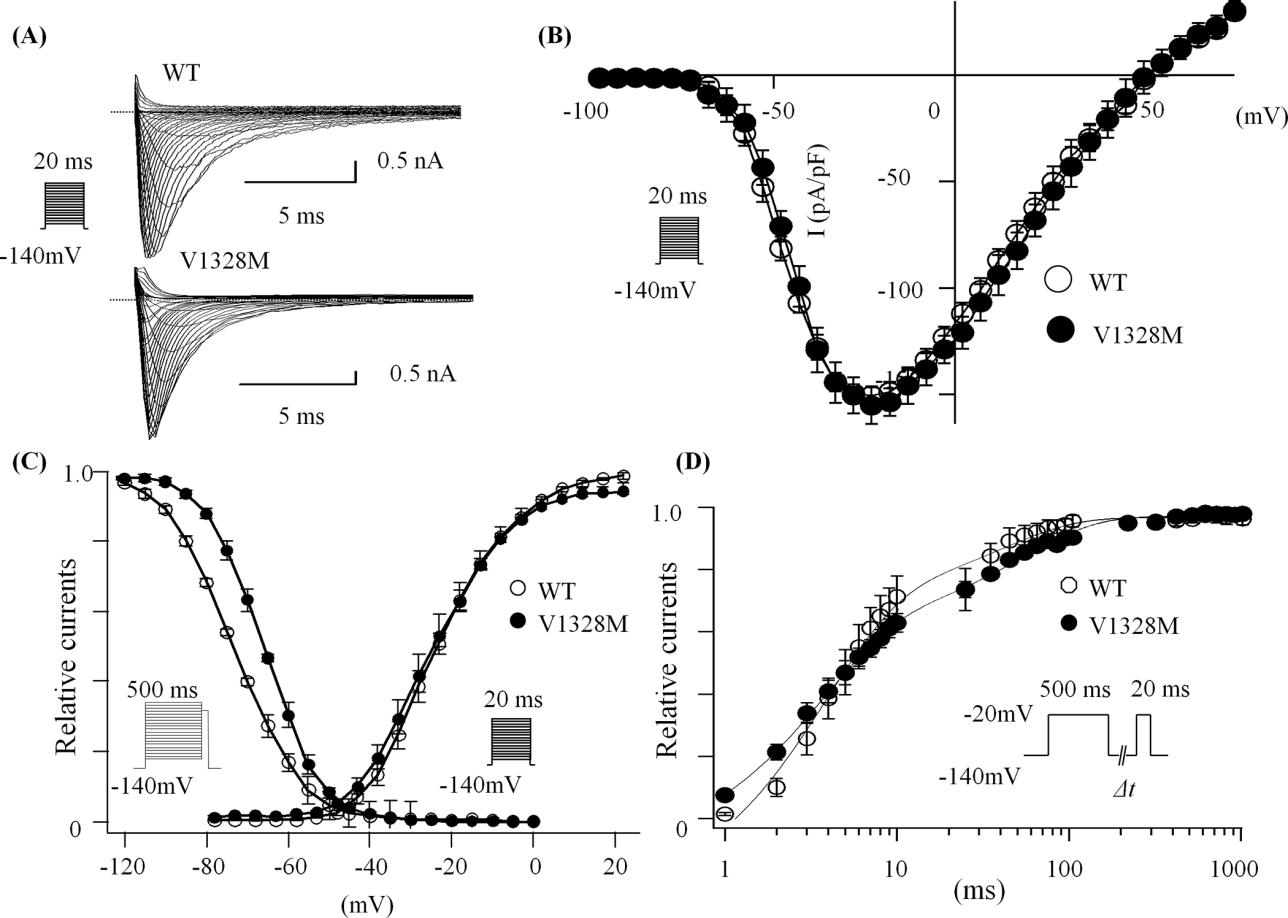
Properties of control and mutant SCNA5 channels expressed in HEK-293 cells. (A) Superimposed whole-cell current traces recorded in response to step changes in membrane voltage. 20 ms were applied in 5 mV increments from -100 to +75 mV from a holding potential (HP) of –140 mV. (B) I-V relationships. (C) Voltage-dependence of peak conductance and steady-state inactivation. Conductance G(V) was calculated by the equation: G(V) = I / (V_m_—E_rev_), where I is the peak currents, E_rev_ is the measured reversal potential, and V_m_ is the membrane potential. Normalized peak conductance was plotted against membrane potentials. Steady-state inactivation was measured using a protocol consisting of a 500 ms pre-pulse ranging from -120 to -20 mV in 5 mV increments followed by a 20 ms testing pulse to -20 mV; HP = -140 mV. Normalized peak currents at test pulses were plotted as a function of membrane potentials. Voltage-dependence of activation and inactivation were fitted with the Boltzmann equation. (D) Recovery from fast inactivation estimated by a double pulse protocol (shown in the inset). The currents at the second pulse were normalized to the currents at pre-pulse and plotted against intervals between the two pulses. The time course was fitted with a sum of two exponentials.

Basal hNav1.5 characteristics of WT-SCN5A and V1328M-SCN5A were studied using the whole-cell patch-clamp method. Fig 4A shows representative whole-cell current traces obtained from HEK-293 cells transfected with WT or V1328M-*SCN5A*. Current densities of WT and the V1328M channels were nearly identical (WT: 163.9 ± 5.9 pA/pF, n = 10; V1328M: 158.9 ± 18.4 pA/pF, n = 10) (Fig 4B). Conductance normalized to peak conductance was plotted against membrane potentials (Fig 4C). The data were fitted with the Boltzman’s equation. Voltage-dependence of activation was not altered in the V1328M mutant (*V*_*h*_: WT, -42.6 ± 1.1 mV, n = 7; V1328M, -40.2 ± 0.8 mV, n = 7). However, voltage-dependence of steady-state inactivation was shifted towards more positive potentials by 7 mV (*V*_*h*_: WT, -100.2 ± 0.8 mV, n = 7; V1328M, -93.1 ± 0.7 mV, n = 7, p <0.01) (Fig 4C and Table 1).

**Table 1:** Parameters of WT and V1328M channels in the absence of drugs.

	WT	V1328M
**Current density (pA/pF)**	(10)	(10)
	163.9 ± 5.9	158.9 ± 18.4
**Activation**	(7)	(7)
*V*_*h*_ (mV)	-42.6 ± 1.1	-40.2 ± 0.8
*k*	8.9 ± 0.3	8.7 ± 0.4
**Steady-state inactivation**	(7)	(7)
*V*_*h*_ (mV)	-100.2 ± 0.8	-93.1 ± 0.7٭
*k*	6.3 ± 0.6	6.7 ± 0.2
**Recovery from fast-inactivation**	(7)	(7)
*τ*_*f*_ (ms)	4.61 ± 0.49	4.51 ± 0.47
*τ*_*s*_ (ms)	53.4 ± 2.4	54.9 ± 2.1
*A*_*f*_	0.83 ± 0.02	0.93 ± 0.02
*A*_*s*_	0.25 ± 0.02	0.18 ± 0.01

*V*_*h*_, represents midpoint voltage of maximal activation/inactivation; *k*, slope factor; *τ*_*f*_ and *τ*_*s*_ the time constants of fast and slow components of recovery; *A*_*f*_ and *A*_*s*_, the fraction of fast and slow components

*p <0.01; numbers in parenthesis, the number of patches.

Recovery from fast inactivation was examined using a double-pulse protocol. Currents during test pulse were normalized to currents during the pre-pulse state and plotted against intervals between the two states (Fig 4D). The data were fitted with a double exponential function. V1328M did not affect the time course of recovery from the inactivation state (n = 7). Table 1 summarizes gating kinetics parameters in the setting of no drugs.

### Tonic block of WT and V1328M-SCN5A by pilsicainide

**Fig 5:**
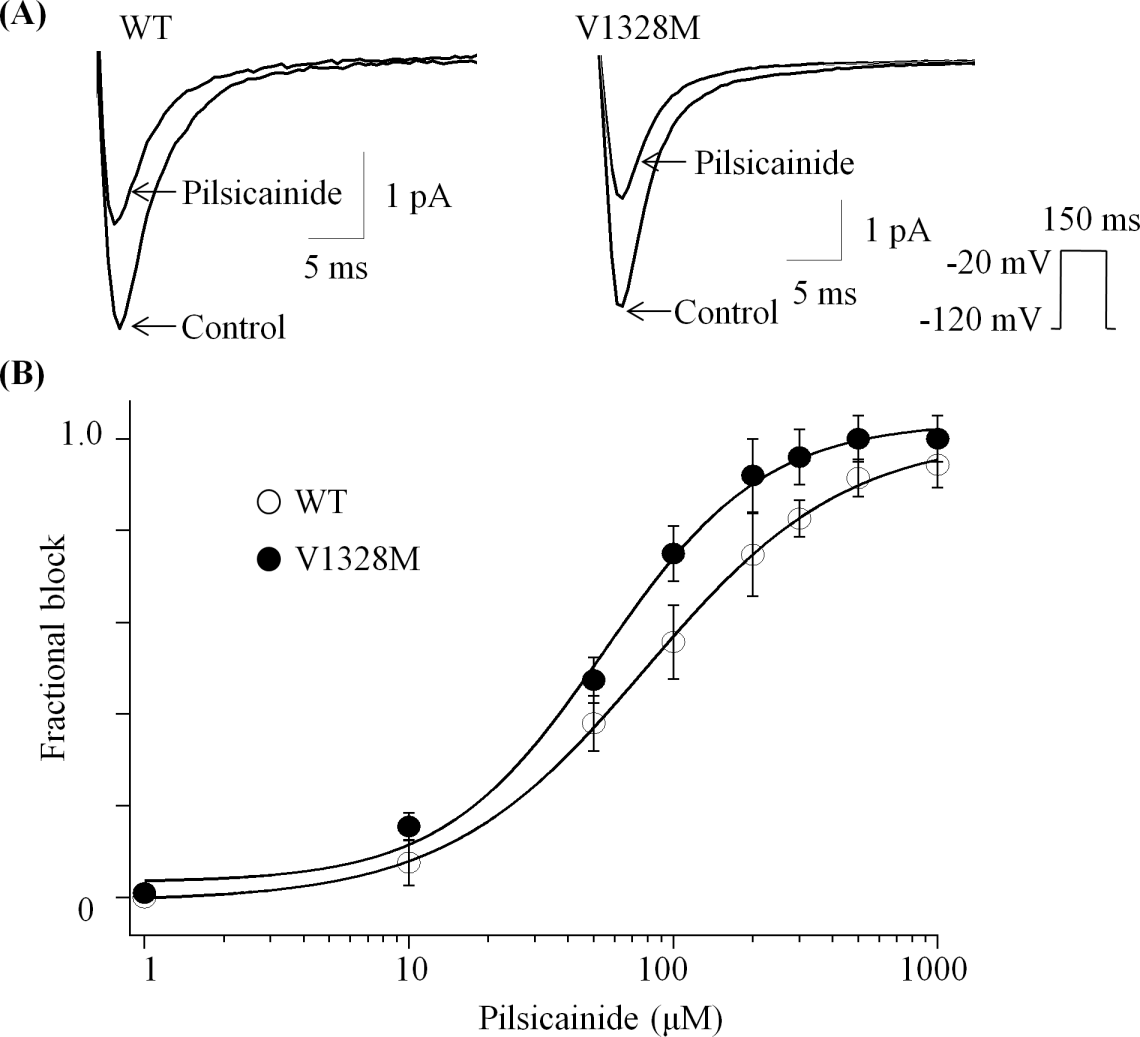
Tonic block of WT and V1328M by pilsicainide. (A) Representative traces in WT and V1328M in the absence or presence of 50 μM pilsicainide. Currents were elicited every 15 seconds by 150 ms pulses to -20 mV from a holding potential of -120 mV. (B) Dose-response relationship of the tonic block. The data were fitted with the Hill equation: y = 1/[1 + (x/*Ki*)^*n*^], where y represents the fractional block; x is the concentration of quinidine; *Ki* is the half-maximal concentration of inhibition; and *n* is the Hill coefficient. Numbers in parentheses are the number of patches.

Tonic block of hNa_v_1.5 by pilsicainide was studied using 20 ms depolarizing voltage steps to -10 mV every 15 s from a holding potential of -120 mV. Fig 5A shows representative current traces in the absence or presence of pilsicainide (50 μM). Fractional block was measured as the ratio of the difference between peak currents in WT and V1328M to peak currents in WT and was plotted as a function of pilsicainide concentration. Pilsicainide (50 μM) exerted similar tonic block in WT and V1328M (% block with 50 μM pilsicainide: WT, 44.2 ± 0.01, n = 6; V1328M, 38.6 ± 0.03, n = 6) (Fig 5B).

### Use-dependent block of WT and V1328M-SCN5A by pilsicainide

**Fig 6:**
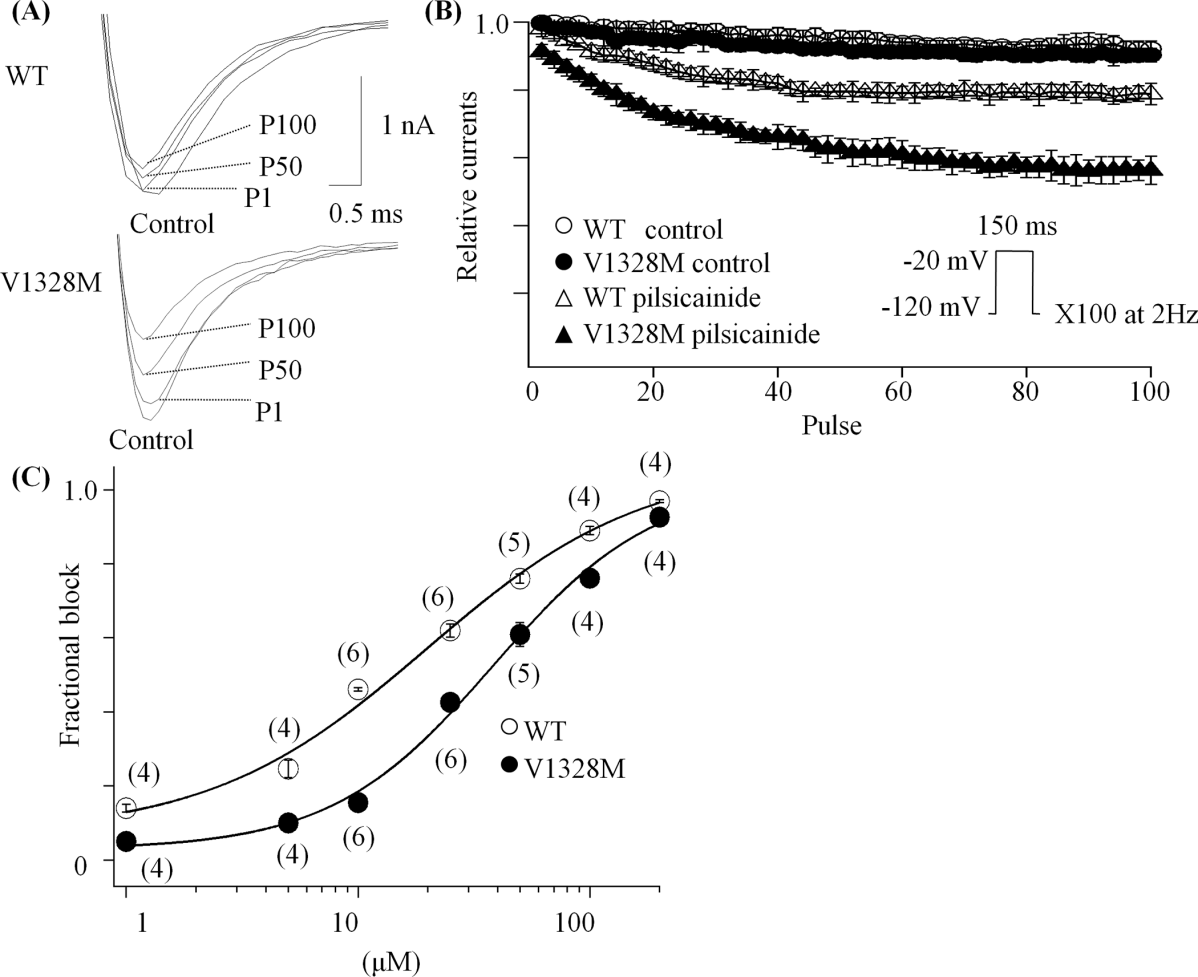
Use-dependent block of WT and V1328M by pilsicainide. (A) Superimposed current traces in the absence or presence of 10 μM pilsicainide at pulse number 1, 50 and 100 (P1, P50 and P100). Currents were elicited every 0.5 second by 150 ms pulses to -20 mV from a holding potential of -120 mV. (B) Normalized currents were plotted against the pulse number. (C) Dose-response relationships of the use-dependent block. Fractional block was estimated at pulse #100 in the absence or presence of the drug. The data were fitted with the Hill equation.

Class I antiarrhythmic agents have been reported to show use-dependent block in voltage-gated sodium channels [14]. Use-dependent block of WT and V1328M by pilsicainide was assessed by application of a 2 Hz pulse train (150 ms at -10 mV) from a holding potential of -120 mV. Fig 6A shows superimposed current traces in WT and V1328M cells in the absence or presence of 10 μM pilsicainide. Currents at each pulse were normalized to the currents at pulse #1 in the absence of the drug and plotted against the pulse number (Fig 6B). Fractional block was estimated as the difference of peak currents at pulse #100 in the presence or absence of the drug. V1328M was found to exhibit more use-dependent block than the WT (% block with 10 μM pilsicainide: WT, 15.5 ± 0.02, n = 6; V1328M, 46.1 ± 0.01, n = 6, *p =* <0.001) (Fig 6C). Using the Hill equation, dose-response relationships were estimated yielding *Ki* values of 35.8 μM and 19.3 μM, and Hill coefficients of 1.4 and 1.1 for WT and V1328M, respectively.

## Discussion

In this study, we report that the novel *SCN5A* mutation, p.V1328M, identified in a patient who presented with drug-induced Brugada-type ECG, can enhance hNa_v_1.5’s response to pilsicainide. Our study also suggests that this *SCN5A* mutation which changes a residue in the cytoplasmic loop, can modify hNa_v_1.5’s response to class IC antiarrhythmic agents that may bind to the extracellular side of the channel.

### Modification of hNa_v_1.5 electrophysiological parameters by V1328M

Electrophysiological data obtained using the patch-clamp method demonstrated that V1328M does not affect sodium current density. On the other hand, V1328M shifts the voltage-dependence of steady-state inactivation toward positive by 7 mV without affecting activation kinetics, with the end result of an increase in channel availability (*i*.*e*., gain-of-function). Interestingly, several variants causing changes adjacent to residue 1328 have been identified in patients with type 3 long-QT syndrome (LQT3) [15–19] and among those variants E1295K and A1330P showed similar biophysical modifications of the Na_v_1.5 as V1328M (*i*.*e*., increase in window currents) [15, 18]. However, our patient did not show LQTS phenotype (QTc = 413 ms) except for being susceptible to VF (i.e., inducible VF). This might be due to the presence of unidentified compensatory mechanisms that can counteract the gain-of-function caused by V1328M. Regardless, it is very unlikely that the gain-of-function in hNa_v_1.5 caused by p.V1328M can result in Brugada phenotype.

### Does modification of hNav1.5 by V1328M account for the observed ECG phenotype?

Approximately a hundred of *SCN5A* mutations were identified in patients with Brugada Syndrome. Electrophysiological studies using cell expression systems and animal models demonstrated that the attenuated sodium channel function caused by these *SCN5A* mutations can result in the loss of the action potential (AP) dome, thereby exaggerating the transmural repolarization gradient [20]. However, the extent of sodium channel modification required to cause a loss in AP dome has not been fully elucidated. Although many *SCN5A* mutations reduce sodium current densities by more than 80%, some of them decrease peak currents by only ~15% (e.g., L567Q and R1023H) [21, 22]. In our patient, V1328M significantly augmented blockage of sodium currents caused by pilsicainide (by 20–30% more compared to WT) within its therapeutic concentration range (10~25 μM; Fig 5C and 5D). Although V1328M might enhance transmural AP dispersion by unidentified mechanisms, it is reasonable to conclude that the pharmacological modification by V1328M, at least in part, accounts for the observed ECG phenotypes.

### Modification of hNa_v_1.5 pharmacokinetics by V1328M

It has been reported that class I antiarrhythmic agents can block sodium channels by plugging their pore from the intracellular side [23]. Studies using site-directed mutagenesis revealed that the hydrophobic residues in the membrane spanning domain DIV-S6 (*e*.*g*., F1764 and Y1771) might serve as common binding sites for these drugs through interaction with the drugs’ hydrophobic moieties [24, 25]. Several studies also indicated that class I antiarrhythmic agents, passing through the channel pore or the plasma membrane, can bind to the residues in pore lining domains [24–26].

Pilsicainide hydrochloride, a derivative of lidocaine, shares a similar hydrophobic structure with drugs QX314 and QX222, the permanently charged lidocaine derivatives. Since most of pilsicainide molecules are in a charged form at physiological pH (pKa = 10.5), it is possible to speculate that pilsicainide may pass through the channel pore and interact with the common binding sites [27].

## Limitations

There are several limitations in our study: (1) Data were obtained using *in vitro* experiments and a non-cardiomyocyte cell line expressing only the exogenous α-subunit of human cardiac sodium channels; (2) Voltage-clamp pulse protocols used in the study were not physiological. However, our study suggests that the subclinical p.V1328M mutation in *SCN5A* might predispose certain individuals to drug-induced Brugada Syndrome. We believe that this genotype-antiarrhythmic relationship could be incorporated in future treatment guidelines of Brugada Syndrome.

## Conclusion

A novel *SCN5A* mutation, p.V1328M, was identified in a patient who developed pilsicainide-induced Brugada-type ST change. In vitro electrophysiological studies demonstrated that p.V1328M can cause gain-of-function in the hNav1.5. p.V1328M was also found to enhance use-dependent block of hNav1.5, which might account for the observed ST elevation. Thus, we conclude that subclinical SCN5A mutations such as p.V1328M could predispose certain individuals to acquired forms of Brugada Syndrome.

## Supporting Information

S1 Fig VF was induced by programmed stimulation. (TIF)

## Abbreviations

PAF: paroxysmal atrial fibrillation
VF: ventricular fibrillation
WT: wild type
