# Supplementary figures and images for: A Novel SCN5A Mutation Associated with Drug Induced Brugada Type ECG
Source: PLoS One. 2016 Aug 25;11(8):e0161872. doi: 10.1371/journal.pone.0161872 (PMC4999187; doi:10.1371/journal.pone.0161872)

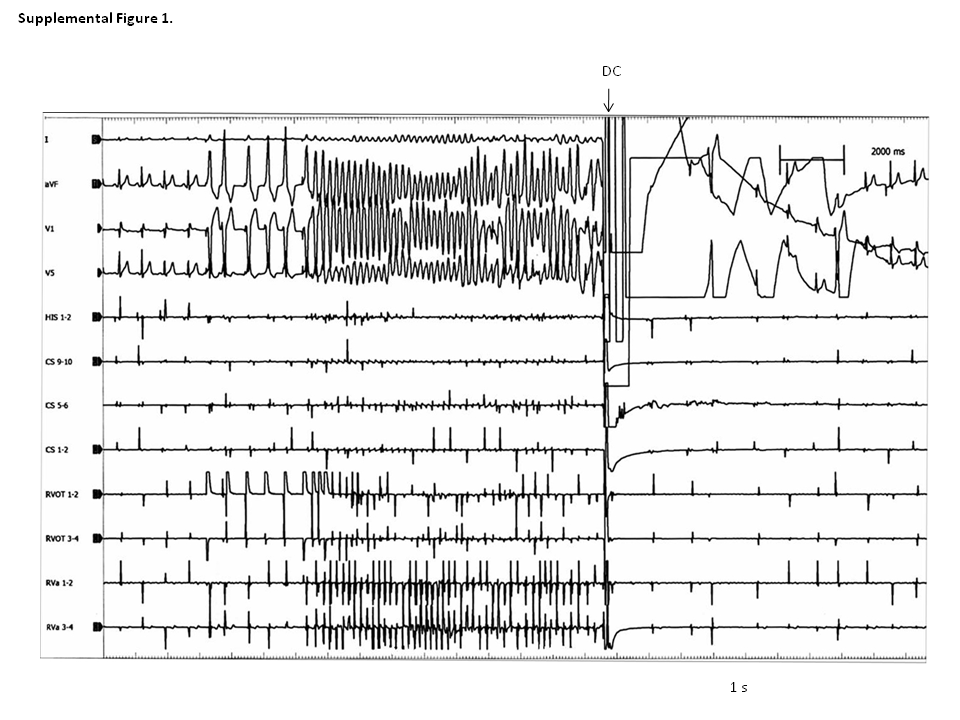

Supplement: S1 Fig — (TIF) [file pone.0161872.s001.TIF]
